# Simultaneous current, force and dissipation measurements on the Si(111) 7×7 surface with an optimized qPlus AFM/STM technique

**DOI:** 10.3762/bjnano.3.28

**Published:** 2012-03-15

**Authors:** Zsolt Majzik, Martin Setvín, Andreas Bettac, Albrecht Feltz, Vladimír Cháb, Pavel Jelínek

**Affiliations:** 1 Institute of Physics, Academy of Sciences of the Czech Republic, Cukrovarnicka 10, 162 53, Prague, Czech Republic; 2 Omicron NanoTechnology GmbH, Limburger Strasse 75, D-65232 Taunusstein, Germany

**Keywords:** AFM, cross-talk, current, dissipation, force, qPlus, STM, tuning fork

## Abstract

We present the results of simultaneous scanning-tunneling and frequency-modulated dynamic atomic force microscopy measurements with a qPlus setup. The qPlus sensor is a purely electrical sensor based on a quartz tuning fork. If both the tunneling current and the force signal are to be measured at the tip, a cross-talk of the tunneling current with the force signal can easily occur. The origin and general features of the capacitive cross-talk will be discussed in detail in this contribution. Furthermore, we describe an experimental setup that improves the level of decoupling between the tunneling-current and the deflection signal. The efficiency of this experimental setup is demonstrated through topography and site-specific force/tunneling-spectroscopy measurements on the Si(111) 7×7 surface. The results show an excellent agreement with previously reported data measured by optical interferometric deflection.

## Introduction

The invention of scanning probe techniques, in particular scanning tunneling microscopy (STM) [1] and atomic force microscopy (AFM) [2], had a tremendous impact on our understanding of the physical, chemical and material properties of surfaces and nanostructures at the atomic scale. STM is based on the detection of the tunneling current between a probe and a sample, and it turned quickly into a standard technique widely used to characterize conductive surfaces and to modify objects at the atomic scale. Unfortunately, the requirement of conductive samples strongly prevents the STM technique from potential applications on nonconductive surfaces, e.g., technologically important oxide materials.

This serious limitation was overcome by the introduction of AFM, which detects forces acting between the tip and the sample. Atomic-scale imaging was achieved later on for both conductors and insulators [3] by means of the so-called static mode. The main drawback of static-mode AFM is the presence of a strong tip–sample interaction, which makes scanning destructive for both the tip and sample, and reliable interpretation of the atomic contrast becomes very difficult. The next milestone in AFM history was the introduction of the frequency-modulation (FM)-AFM technique by Albrecht and co-workers [4]. By applying this method Giessibl demonstrated the possibility of achieving true atomic resolution on the prototypical Si(111) 7×7 surface [5]. Among others, this seminal work initiated a fast progression of the FM-AFM technique over the past decade [6–7].

At the beginning, mainly silicon-based cantilevers oscillating with large amplitudes (tens of nanometers) were used, because they possess the important oscillation stability [8–10]. The key factor to achieve atomic resolution is the proper choice of several parameters, for example, the spring constant and the oscillation amplitude (see Table I in [11]). Theoretically, the optimal signal-to-noise ratio (SNR) is achieved at a value of the oscillation amplitude that is comparable with the characteristic decay length (κ*_F_*) of the forces responsible for imaging. Thus, the optimal oscillation amplitude should be on the order of a few angstroms or even less. Furthermore, an additional benefit of a small oscillation amplitude is the reduction of the sensitivity to contributions from long-range forces. Also large-amplitude operation significantly decreases the measured value of the time-averaged current and subsequently reduces the sensitivity in detection of the tunneling current. Therefore the application of small amplitudes in simultaneous AFM/STM experiments seems to be a natural choice.

Consequently, a new kind of sensor was introduced, based on a quartz resonator, into the field of FM-AFM. So far, the most popular and reasonable way to reach the desired small amplitudes is to replace the microfabricated (Si) cantilevers by stiff, piezoelectric quartz tuning forks similar to those used as frequency references in watches. The configuration when one of the prongs is attached to a solid substrate and the free prong acts as a cantilever with the capability of self-sensing, is called qPlus, named by Giessibl [12]. One of the largest benefits of this design is that it has nearly the optimal stiffness for the operation of FM-AFM at low amplitudes while keeping the force sensitivity high enough [13]. Not surprisingly, the qPlus design presented high potential for outstanding atomic-scale imaging from its early stages [14]. In addition, the parts of the qPlus sensor are large enough for assembly of the sensor simply by hand. Let us note that using a length-extensional resonator is another interesting alternative to the qPlus configuration [15–16]. The comparison of their performance is still an open issue in the community [13].

Probably the first measurement of forces acting between the tip and the sample during STM scanning was performed by Dürig et al. [17] already in 1986. Further attempts to perform simultaneous STM and AFM measurements by FM-AFM [18–20] appeared almost a decade ago. Recently, there has been an increasing number of successful simultaneous AFM/STM measurements with coated Si-cantilevers [21–24], qPlus sensors [25–28] and length-extensional quartz resonators [16,29]. The possibility of measuring the interaction forces simultaneously with the flow of electrons between the tip and the sample opens a new horizon in the understanding of elemental processes of the electron transport on surfaces [30] and in clarifying the relationship between the short-range force and the tunneling current in metal contacts [31–32].

Unfortunately, in the case of quartz-based sensors with self-sensing, the presence of the tunneling current may introduce an undesired interference (cross-talk) between the current and the deflection channel. Therefore special attention has to be paid to minimize the impact of this phenomena to a negligible level. Albers et al. [33] already mentioned a kind of coupling of the tunneling current and used, as a solution, a separate wire for the current measurement.

In this paper, we investigated the origin of the coupling between the deflection and the tunneling-current channel. As a result, we show that the cross-talk is a result of the speed limit of the current-to-voltage converter used for detection of the tunneling current and the stray capacitance between the internal connections of the microscope. Based on our findings, we made some modifications of the sensor design and of the internal wiring too. Simultaneous STM/AFM measurements on the Si(111) 7×7 surface with the modified setup were carried out to prove that the cross-talk has no significant impact on the measured quantities. Simultaneously measured force, tunneling current and dissipation are compared to theoretical predictions [34] and with measurements of the optical interferometric deflection [21].

## Experimental

### General description

The measurements were performed at room temperature with an Omicron VT XA qPlus AFM/STM system operating at a base pressure below 1 × 10^−10^ mbar. In this experimental setup, the tunneling current is acquired with an in vacuo preamplifier floating at the potential of the bias voltage and the sample holder is grounded. NanoSurf EasyPLL is used for the FM demodulation and the Omicron MATRIX control system for the data acquisition. The qPlus sensors were built from commercially available tuning forks from Micro Crystal, originally packed in the SMD package MS1V-T1K. The original tuning fork was shortened in order to reach higher sensitivity (charge produced by deflection) [35], which allows us to reach lower amplitudes. The interaction force between the tip and surface atoms was calculated from the measured frequency-shift data by means of the Sader formula [36]. The tunneling current *I*_t_ was calculated from the time-averaged tunneling current <*I*_t_> by using a similar approach [37].

### Cross-talk between the deflection and the tunneling-current channel

The aim of this section is to discuss the basics of the so-called cross-talk phenomenon, in which interference between the current and deflection channels leads to undesired modulation of the tuning-fork motion. First, we will demonstrate how the modulation of the tunneling-current signal due to dynamic motion of the probe may affect the functionality of the current-to-voltage converter. In particular, we will discuss conditions under which the virtual ground is no longer constant. Combination of the oscillating ground potential with the presence of a stray capacitance between the wires connecting the electrodes to operational amplifiers may induce a current between the channels. This current leads to artificial modulation of the detection channel resulting in the so-called cross-talk phenomena. We will show, that the cross-talk is controlled by three parameters: (i) The resonant frequency *f*_0_ of the fork; (ii) the stray capacitance *C*_c_ and (iii) the maximum amplitude of the modulation 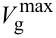 of the virtual ground potential. The last parameter is a function of the oscillation amplitude *A* and the characteristic decay length of the tunneling current κ*_I_* and depends on the characteristics (mainly on the slew rate) of the preamplifier.

**Figure 1:**
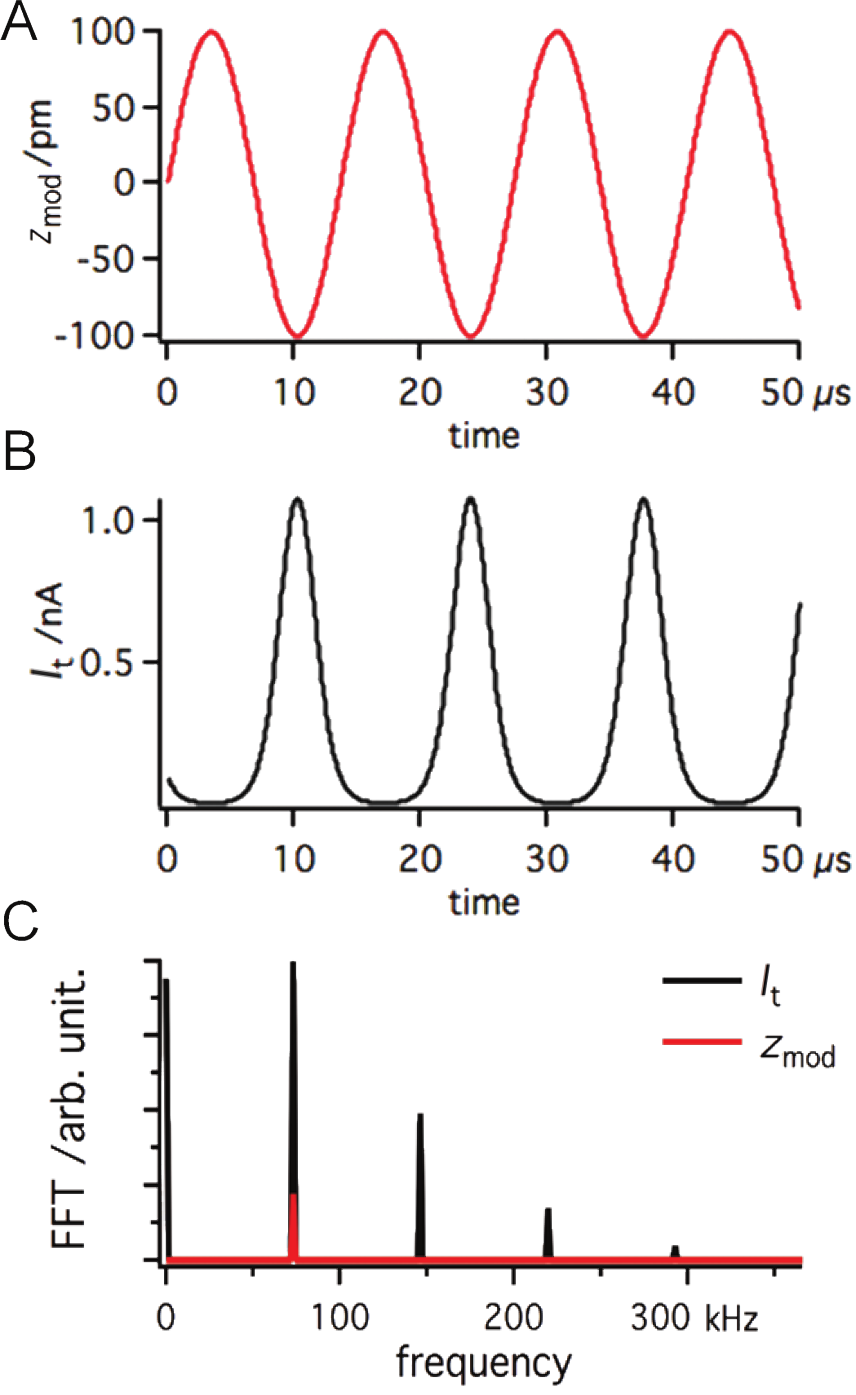
The effect of *z* modulation (A) on the tunneling current (B). *a*_mod_ = 0.1 nm and *f*_0_ = 73180 Hz; *I*_t_ calculated by using *I*_t_(*z*) = *I*_0_e^−2κ^*^z^* where κ*_I_* = 11.9 nm^−1^ and *I*_0_ = 0.1 nA. In order to see better, the frequency distribution FFT is also shown (C).

In FM-AFM mode, the sensor oscillates with the resonant frequency *f*_0_. Upon a decrease of the tip–sample distance the value of *f*_0_ is changed by Δ*f* due to forces acting between the probe and the sample. If the tip and the sample are conductive, a tunneling current *I*_t_ can be detected. The impact of the modulation of the tip–sample distance on the tunneling current is shown in Figure 1. Since the tunneling current depends exponentially on the tip–surface separation *z* as *I*_t_(*z*)*=I*_0_e^−2κ^*^z^*, the harmonic modulation produces sharp peaks in the current signal (Figure 1B). As a consequence, the frequency spectrum of the tunneling current shows higher harmonics of the modulation signal (Figure 1C).

**Figure 2:**
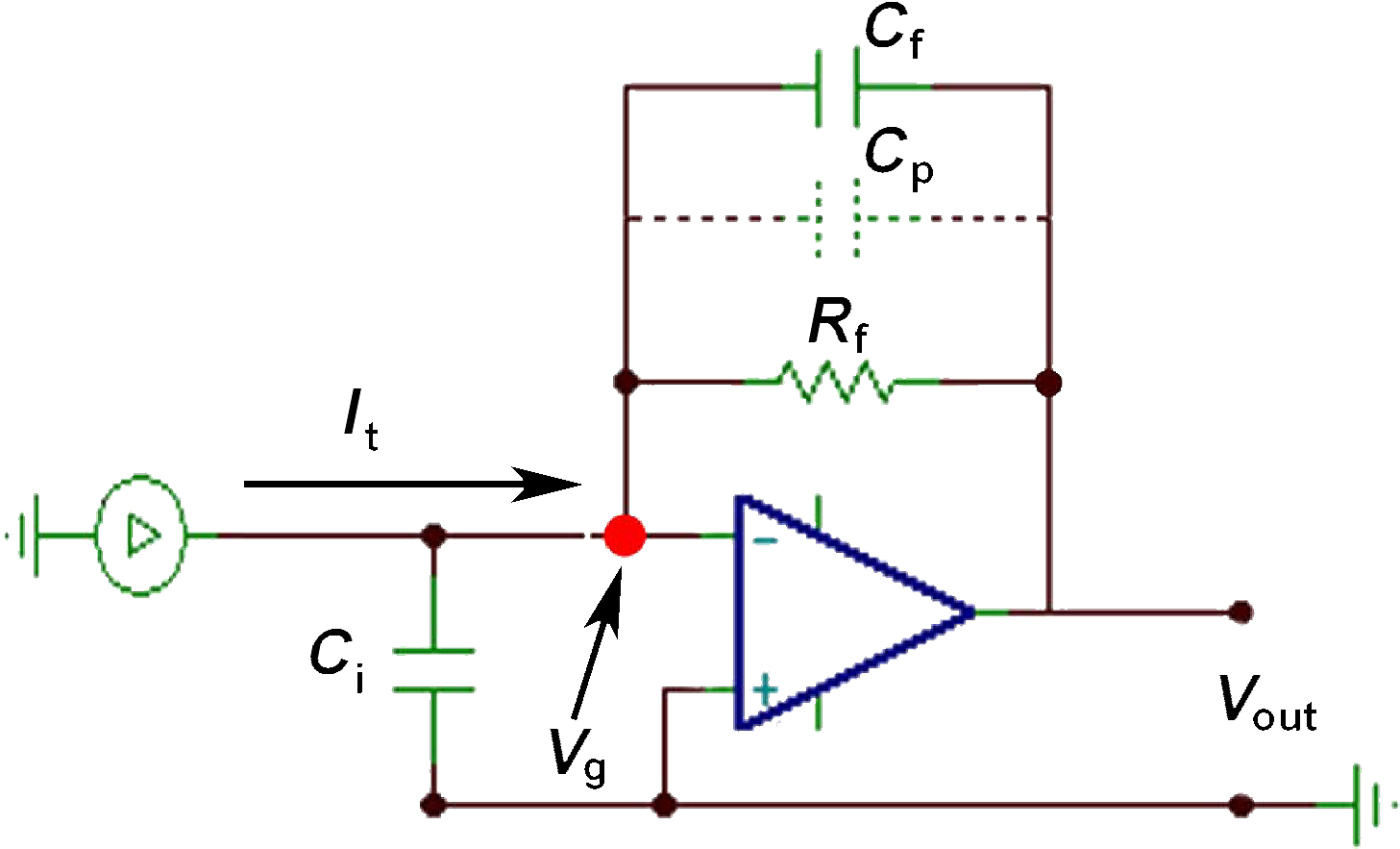
Circuit diagram of a current-to-voltage converter (IVC) where *R*_f_ is the feedback resistance with the parallel capacitance *C*_f_. *C*_i_ is the input capacitance (in addition to the one of the amplifier). *C*_p_ represents the parasitic capacitance of the feedback resistor. The input of the operational amplifier floats at the virtual ground potential (*V*_g_).

The tunneling current *I*_t_ is converted to a voltage signal *V*_out_ with the current-to-voltage converter (IVC). The circuit diagram of an IVC is presented in Figure 2, where *R*_f_ is the feedback resistor with the parallel capacitance *C*_f_ and *C*_i_ represents the input capacitance caused by cabling. The current passing through the feedback resistor R_f_ induces a voltage drop that is equal to the value of *R*_f_*I*_t_. Due to the potential difference between the input terminals, the operational amplifier (OPA) will change its output voltage *V*_out_, compensating the voltage drop to ensure zero potential-difference between the input terminals. Because the inverting input is kept at the ground potential this terminal is called the virtual ground. The output voltage correlated with the tunneling current is *V*_out_ = −*R*_f_*I*_t_.

When working with small values of the tunneling current (on the order of nA) the feedback resistance *R*_f_ must be high enough to achieve a reasonable value for the output voltage. However there is a side effect to high-gain operation. The frequency response is strongly reduced as the gain is inversely proportional to the bandwidth. In such a regime, the feedback capacitor *C*_f_ plays an important role in the circuit reducing the gain at high frequencies (i.e., eliminates instabilities and prevents self-oscillations). In a real circuit, the parasitic capacitance *C*_p_ across the large-feedback resistor *R*_f_ (≈100 MΩ) is in the range of a picofarad, which fully covers the function of the feedback capacitor *C*_f_. Therefore, we will consider only the parasitic capacitance *C*_p_ in the rest of the discussion.

**Figure 3:**
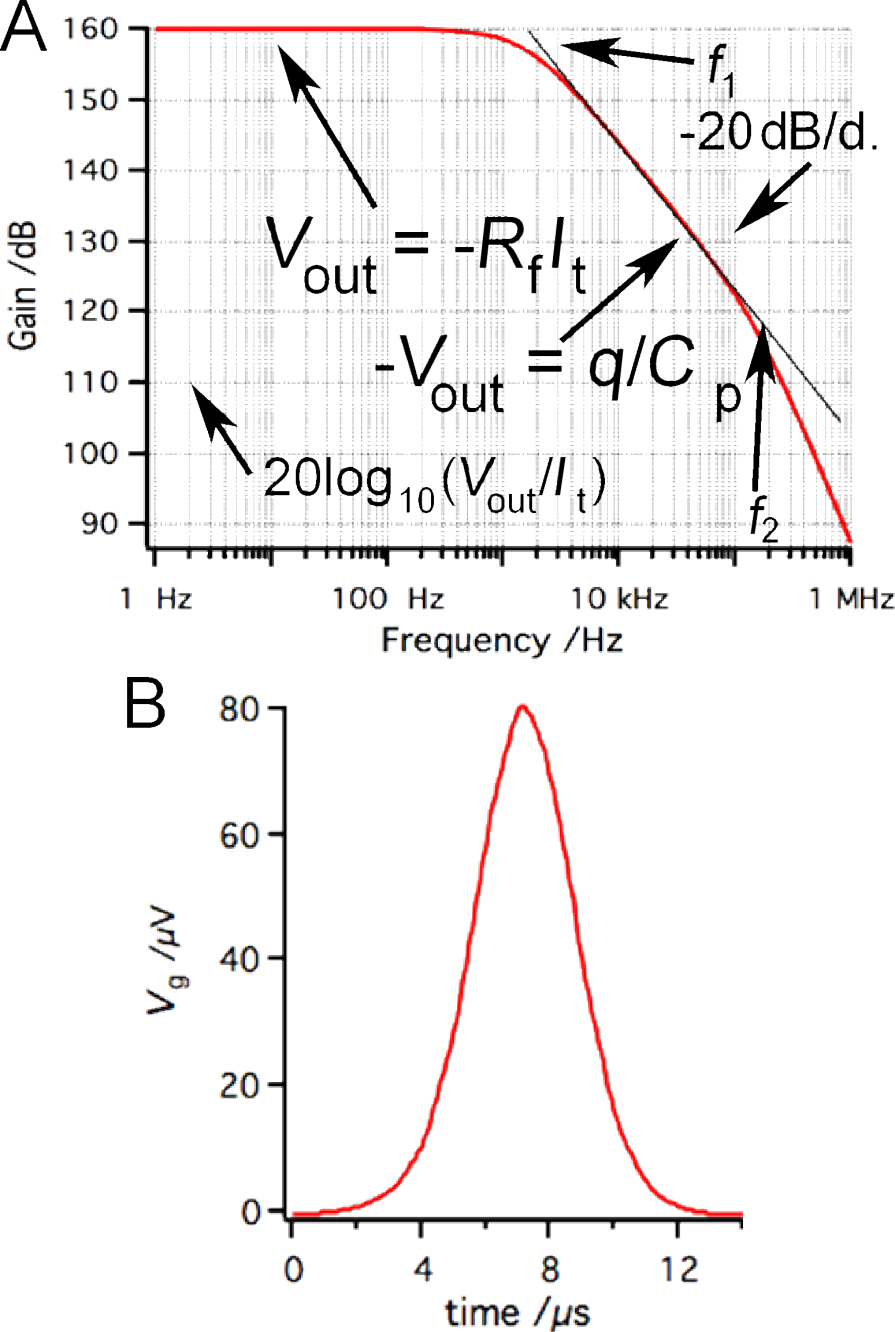
(A) Frequency response of the IVC presented in Figure 2. The following parameters were used to simulate *R*_f_ = 100 MΩ, *C*_p_ = 1 pF and *C*_i_ = 10 pF. (B) The effect of the tunneling current presented in Figure 1 on the virtual ground.

For circuit analyses we performed numerical simulations with the SPICE-based analog simulation program TINA-TI [38]. The frequency response of the IVC is shown in Figure 3A. For calculations we used the macro model of Op111 with parameters *R*_f_ = 100 MΩ, *C*_p_ = 1 pF and *C*_i_ = 10 pF. The parameter *C*_i_ corresponds to the capacitance of the ≈10–15 cm long coaxial cable (depending on the exact type of the cable) making the connection between the tip and the input of the OPA [39].

As already mentioned, the voltage drop will appear on the inverting input of the OPA. The OPA will counteract by producing the same voltage on the output, but with opposite sign to keep the differential voltage at zero between the input terminals. As it can be seen from Figure 3A the output voltage *V*_out_ varies with the frequency. In terms of currents, the output voltage can be better expressed as −*R*_f_*I*_t_/(1 + 2π*fR*_f_*C*_p_) [40]. At small frequencies, the term 2π*fR*_f_*C*_p_ is negligible and the original expression for the output voltage *V*_out_ = −*R*_f_*I*_t_ is recovered. For the frequencies higher than the first frequency pole *f*_1_ = 1/(2π*R*_f_*C*_p_) (in our case *f*_1_ = 1.6 kHz) the amplifier gain drops −20 dB/decade, being proportional to 1/*f*. In this regime, the amplifier behaves as an integrator circuit and the value of *C*_p_ becomes dominant. The voltage at the capacitor is equal to the charge *q* on the capacitor divided by its capacitance, therefore *V*_out_ = *q*/*C*_p_. Because the output voltage *V*_out_ is proportional to the charge, it is also called a charge amplifier. The charge amplification breaks at the second pole in the frequency response (*f*_2_) which is around 110 kHz in this particular example setup.

The optimal function of the IVC is guaranteed as long as the value of the virtual ground potential *V*_g_ is held at the ground potential. *V*_g_ is kept constant by varying the output voltage *V*_out_. The slew rate of the OPA determines the maximum speed at which the output voltage can change. For sinusoidal changes given by 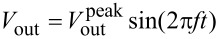, the slew rate must exceed

[1]
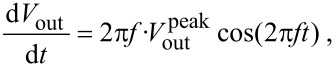


with the maximum value at *t* = 0:

[2]
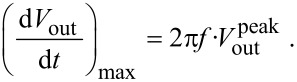


For the resonant frequency of the tuning fork (73180 Hz) and the output voltage of 1 V, the maximum speed (d*V*_out_/d*t*) is 0.46 V/μs. The maximum slew rate of Op111 is 2 V/μs which means that optimal operation of the IVC is ensured for output amplitudes <4.3 V at the given frequency. As was already shown in Figure 1, the tunneling current during dynamic AFM measurements contains much higher frequency components than the resonant frequency of the tuning fork. Therefore the OPA may not be able to keep the virtual ground (gap voltage) constant with high precision, due to the speed limit of the amplifier. When data from Figure 1 are used for simulation of the circuit function, *V*_g_ shows oscillations with peak amplitudes 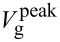 ≈ 80 μV.

**Figure 4:**
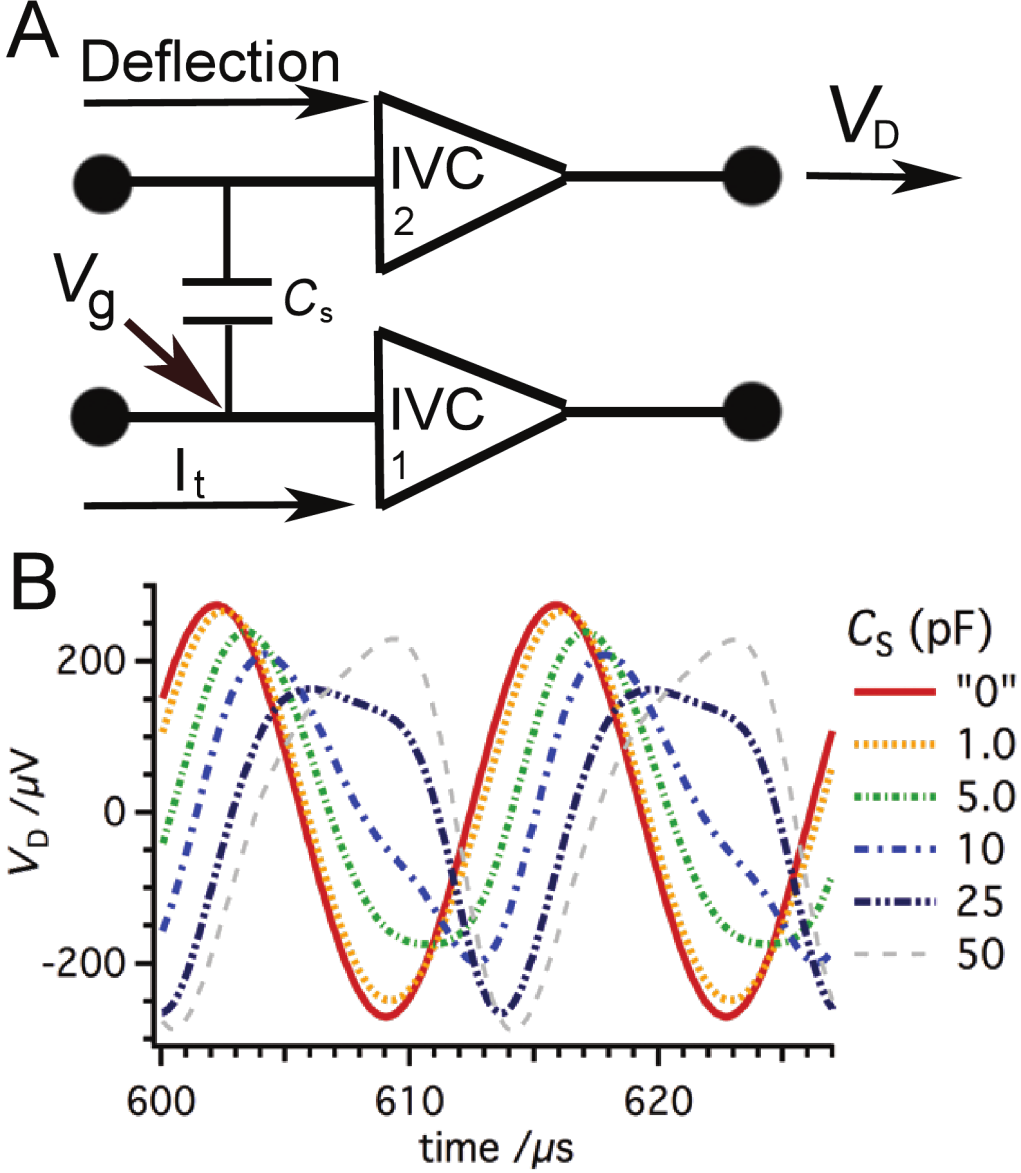
(A) The coupling between the deflection and the tunneling current channel is established by the stray capacitance *C*_s_. To show the effect of the cross talk, signals shown in Figure 1 were used as input values for the amplifiers. (B) The signal in the deflection channel is altered significantly at the output *V*_D_ for *C*_s_ values exceeding 1 pF.

The modulated potential in the current channel may interfere with the input signal of the deflection channel. The coupling of the channels is driven by the stray capacitance *C*_s_ (Figure 4). To demonstrate how the crosstalk affects the deflection signal, we analyzed the configuration shown in Figure 4. To simplify the electric circuit, identical amplifiers were used in both channels (Figure 2). For the given resonant frequency *f*_0_ = 73180 Hz the amplifier operates in the charge amplifier regime (Figure 2). To simulate the output from the deflection channel close to our experimental conditions, 3 μC/m sensitivity was used for the sensor [13]. The transmitted signals, shown in Figure 1, were used as input values of the amplifiers in our simulation. Using 100 pm deflection amplitude with a capacitance *C*_p_ of 1 pF we obtain an output voltage of approx. 300 μV.

If the input terminal of the amplifier in the deflection channel is held at the ground potential, the current 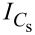 due to the stray capacitance between the channels can be expressed as

[3]
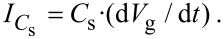


Hence the maximum current 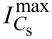 is defined (using Equation 2 for *V*_g_ instead of *V*_out_) as

[4]
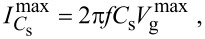


where 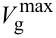 can be estimated by circuit simulations. From Equation 4 we immediately see that the maximum current 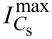 (or in other words the degree of the cross-talk) depends on the value *C*_s_ as well as on the resonance frequency *f*_0_ and the maximum amplitude of the ground potential oscillation 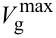. The magnitude of 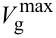 depends also on the frequency *f*_0_, the oscillation amplitude A and the characteristic decay length of the tunneling current κ*_I_*. Therefore the crosstalk can be enhanced when the sensor is operated at high frequencies. Figure 4 shows that the signal in the deflection channel appears significantly altered at the output, *V*_D_, for *C*_s_ values exceeding 1 pF. In the case of a stray capacitance of 5 pF the crosstalk causes a decrease of the initial value of *V*_D_ from 273 μV to 250 μV with a 6.8° phase shift. Note here, that by inverting the sign of the bias voltage the result will be different and even larger oscillation signals can be detected.

**Figure 5:**
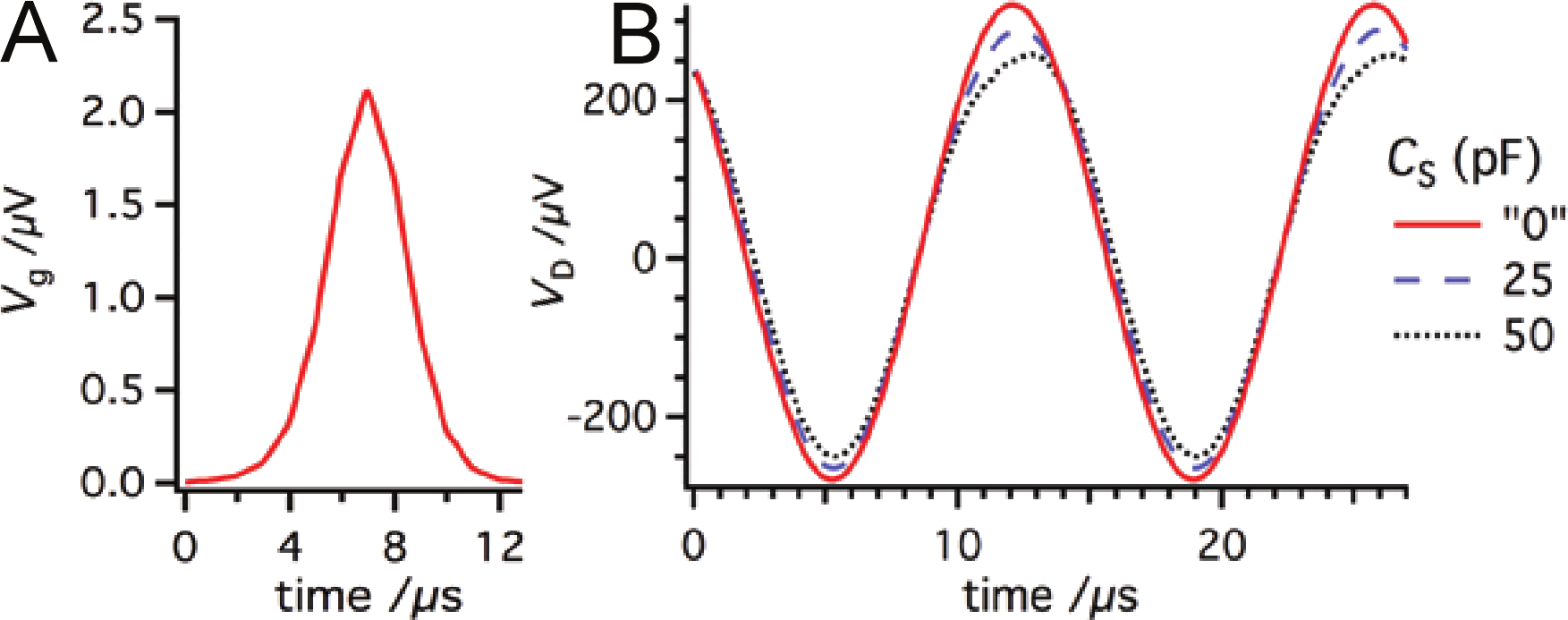
(A) If the slow Op111 is replaced by a faster OPA, Op637, the modulation of the virtual ground 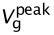 can be significantly reduced (by a factor of 40). (B) Consequently, the signal in the deflection channel is altered at much higher *C*_s_ values.

Together with *C*_s_, the crosstalk depends also on the speed of the amplifier response. It was shown that the virtual ground is modulated when the amplifier response is too slow. The same analysis was carried out with Op637 instead of Op111. The Op637 has a much higher slew rate (≈50 times). The results show that the modulation of the virtual ground is reduced by a factor of 40 (Figure 5A). As a consequence, the crosstalk appears at much higher values of *C*_s_ (Figure 5B).

In conclusion, we showed that the crosstalk between the current channel and the deflection depends mainly on two parameters: (i) The stray capacitance *C*_s_ between the channels; (ii) the resonance frequency *f* of the sensor. The cross-talk alters the detected oscillation amplitude and its phase. The amplitude regulator tends to suppress the artificial oscillation amplitude leading to the appearance of a “dissipation” signal. This can take both positive and negative values. Finally, special attention has to be paid when the tuning fork is used at higher harmonics or higher flexural modes, because the harmonic modulation of the tunneling current can appear in the deflection signal due to the coupling between the channels.

### The prevention of the cross-talk phenomena

In a joint project with Omicron Nanotechnology, we evaluated the crosstalk in the qPlus sensor. We suggested several improvements in order to keep the capacitive couplings as low as possible. First, we modified the construction of the sensor. Originally, one of the electrodes of the tuning fork was connected to the deflection amplifier and the second electrode was used for detection of the tunneling current. The tip was glued directly to the electrode. This arrangement of electrodes can lead to self excitations by the AC component of the virtual ground potential at the *I*_t_ detection path. The capacitance between the electrodes of the tuning fork acts as a coupling capacitor.

**Figure 6:**
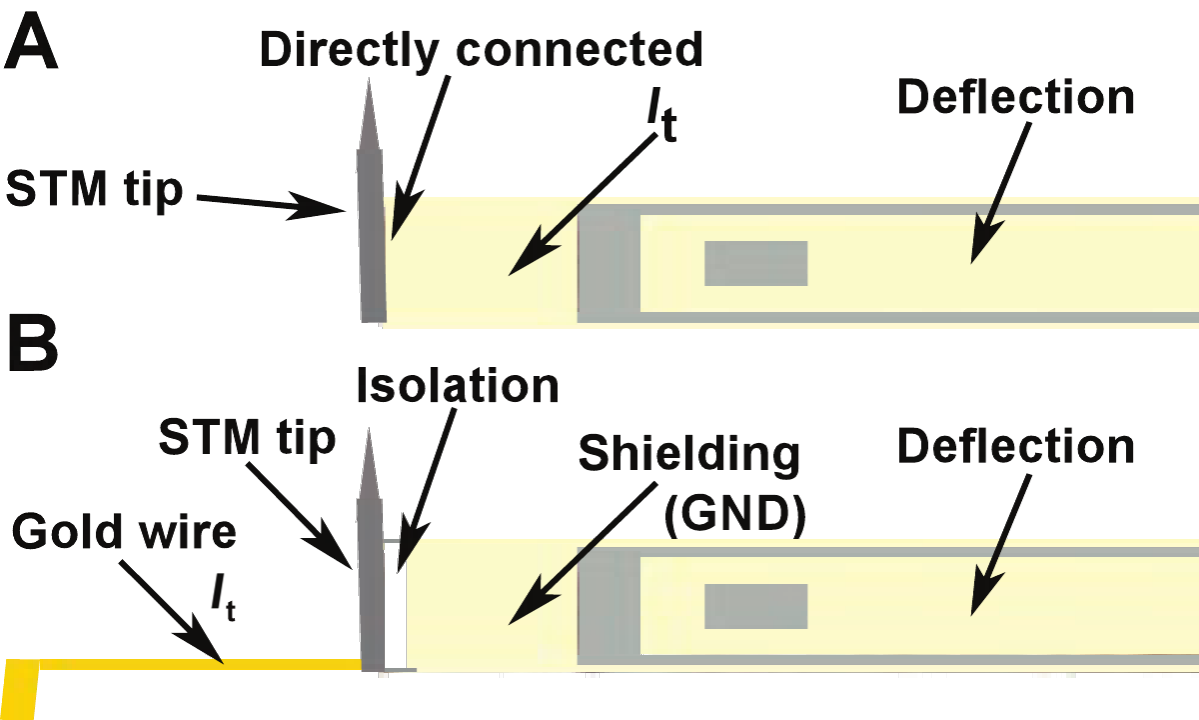
(A) Original connections in which one of the electrodes of the tuning fork was connected to the deflection amplifier and the second electrode was used for detection of the tunneling current. (B) To reduce the capacitance between the channels and eliminate any possibility of self-excitation, the tip is connected by a separated gold wire to the IVC and the electrode originally used for the tunneling current is now grounded. The wire and the tip is electrically isolated from the rest of the fork.

In the new sensor design, the tip is connected with a separate wire (0.25 μm gold) to the OPA for the current channel. The measurement of the tunneling current by means of a separate wire was also reported by other groups [33,41–42]. The electrode originally used for detection of the tunneling current is grounded to create a shielding electrode (Figure 6). The gold wire and the tip on the active prong have to be electrically isolated from the quartz of the prong to avoid self-oscillations of the sensor.

We found that the original ceramic support of the tuning fork with printed wiring increases the capacitance between the tunneling current and the deflection channels. We replaced the ceramics with a metal plate connected to the ground potential. The electrodes of the tuning fork and the tip itself are directly connected to the connector pins. The metal plate now works as an extra shielding between the pins used to connect the current and deflection channels. The modified wiring on the ceramic support, with grounded metal plates on both sides, shows similar electrical properties to those expected for a fully metallic support. Beside the modifications in the sensor design, we replaced the internal coaxial cable making connection between the tip and the tunneling-current amplifier with a double-shielded one, and also the sensor reception stage was altered to reduce the stray capacitance even further. Moreover, as was already mentioned, the sensitivity of the sensor can be increased by shortening the tuning fork. The higher deflection signal reduces the impact of the cross-talk at a given amplitude compared to sensors having the original length, and lower amplitudes can be reached. Let us note that collecting the tunneling current on the sample side with carefully designed internal wiring can be an alternative option for several microscopes.

## Results and Discussion

### Force and tunneling current

**Figure 7:**
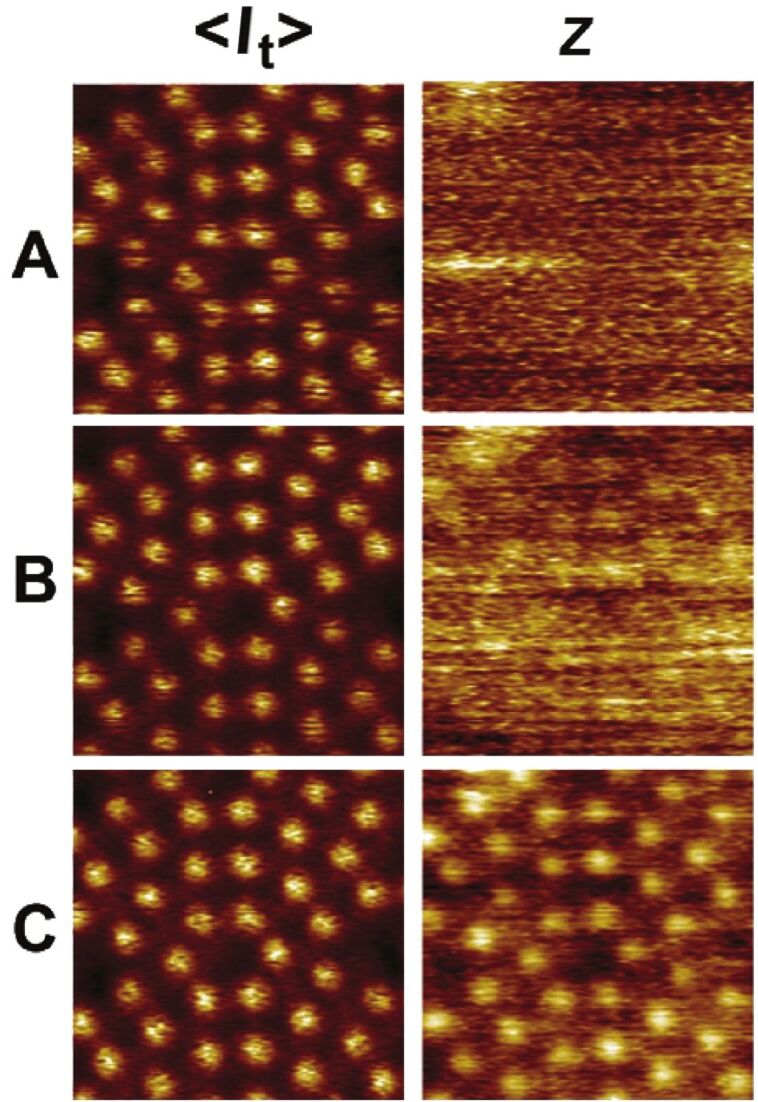
A set of constant-frequency-shift maps (*z*) and simultaneously recorded average-tunneling-current maps (<*I*_t_>). The frequency-shift setpoints for topographic imaging are (A) −35 Hz, (B) −40 Hz and (C) −45 Hz.

We performed simultaneous STM/AFM measurements on the Si(111) 7×7 surface using our modified sensor. The measurements were performed in the constant frequency shift mode at room temperature. To compensate for long-range electrostatic forces, the bias voltage was adjusted to the minimum of the Kelvin parabola (generally about +0.4 V). Figure 7 shows a set of images of the average tunneling current <*I*_t_> and topography at a constant frequency shift (*z*) for decreasing tip–sample separation. While we were unable to observe any atomic contrast in the topography signal at Δ*f* setpoints above about −35 Hz (Figure 7A and Figure 7B), the atomic contrast in <*I*_t_> was already achieved. Upon approach of the tip further towards the sample, the onset of the short-range chemical force *F*_SR_ is reached and the atomic contrast in the *z* map appears. When the setpoint Δ*f* is tuned to more negative values, the atomic corrugation induced by the chemical interaction [43] between the tip apex and the adatoms becomes larger.

**Figure 8:**
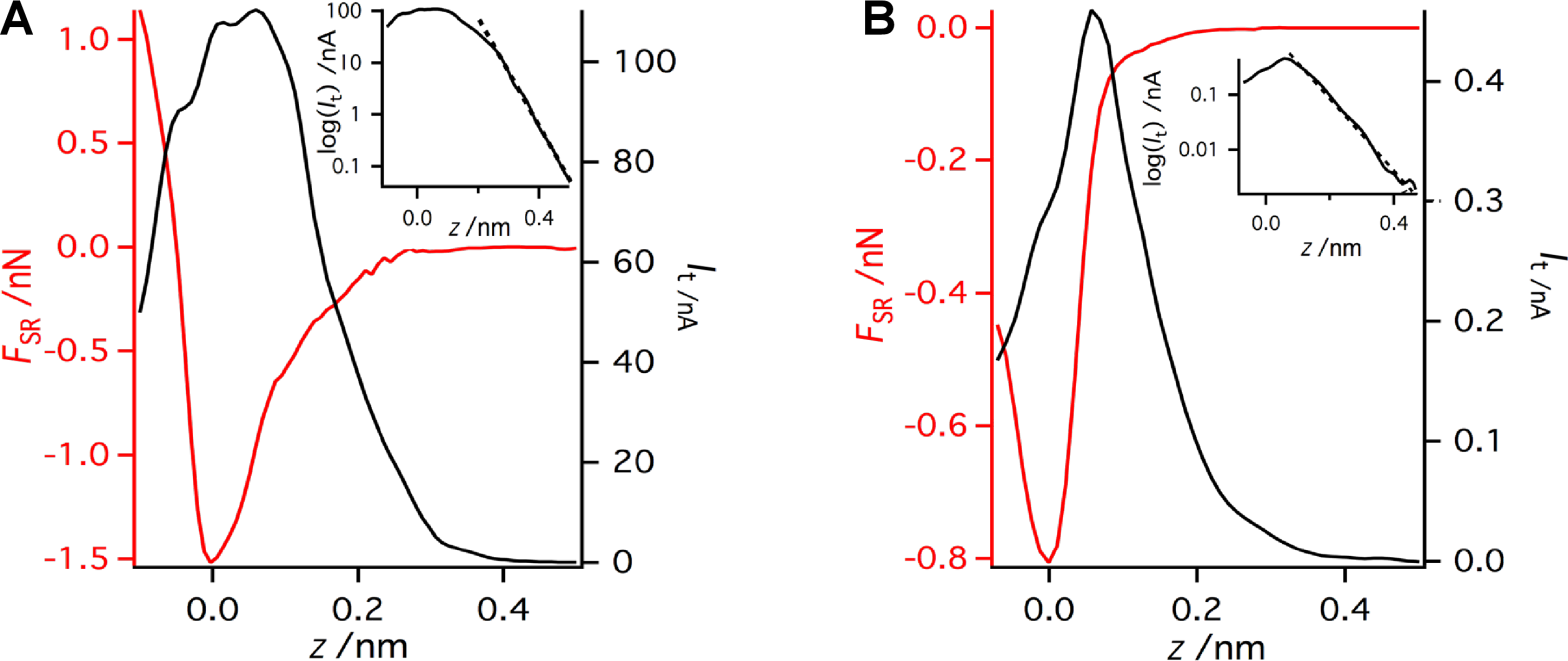
Two typically observed profiles of the dependence of the short-range interaction force (*F*_SR_) and the tunneling current (*I*_t_) on the tip–sample separation (*z*) with a logarithmic plot of *I*_t_ in the inset. The first tip termination (A) presents a much stronger attractive short-range interaction than the second (B), and in addition the tunneling current is much smaller in case (B). The acquisition parameters are *f*_0_ = 73180 Hz, *a* = 0.215 nm, *k* = 3750 N/m, and *V*_bias_ = 0.4 V.

In addition, we performed site-specific point spectroscopy [44–45] above Si adatoms. Note that the spectroscopy curves shown in Figure 8A were obtained with a slightly different tip than the maps in Figure 7. To obtain the bare short-range force above an adatom, we subtracted the long-range component of the force measured above the corner hole site. The dependence of the short-range chemical force and the tunneling current on the tip–sample distance is plotted in Figure 8A.

For this particular tip, the short-range force maximum reached 1.5 nN. Both the tunneling current and the short-range force show an exponential dependence, *A*(*z*) = *A*_0_e^−2κ^*^z^* where *A* stands for *I*_t_ or *F*_SR_, on the tip–sample distance *z* at large distances (for *z* > 0.24 nm for *I*_t_). We also estimated the characteristic decay lengths of the tunneling current κ*_I_* = 11.9 nm^−1^ and the short-range force κ*_F_* = 6.3 nm^−1^. Comparing the characteristic decay lengths κ*_I_* ≈ 1.89 × κ*_F_*, we immediately find that the tunneling current is proportional to the square of the short-range force (*I*_t_ = 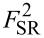). Note that this relation corresponds to the interaction between two localized states degenerate in energy, as was recently predicted theoretically (see a related discussion in [34]).

For distances *z* smaller than 0.24 nm, the tunneling current is no longer an exponential function of the distance *z*. It drops significantly due to the substantial modification of the atomic and electronic structure of the surface dangling-bond state [46]. The drop occurs close to the setpoint, at which the short-range force reaches the maxima. Our spectroscopic data agree very well with similar measurements by means of the beam-deflection method [21].

Additionally, we repeated the spectroscopy measurement with the same sensor but with a different tip apex. The tip change was induced by applying a combination of *z* pulses and voltage pulses. The obtained data show (Figure 8B) a significant reduction of the force maximum of the short-range force *F*_SR_ ≈ 0.8 nN. In the weak-interaction regime (here *z* > 0.07 nm), the exponential dependence is presented. However, the characteristic decay length of the tunneling current κ*_I_* = 7.6 nm^−1^ decreases while the decay length of the short-range force increased to κ*_F_* = 6.9 nm^−1^. The ratio between the characteristic decay lengths is now κ*_I_* ≈ 1.10 × κ*_F_*. Therefore, in this particular case the tunneling current *I*_t_ is closely proportional to the chemical force *F*_SR_, as has been observed experimentally [32] and predicted theoretically [32,34].

### Dissipation signal

The appearance of the dissipation signal and its origin in the FM-AFM experiment has received a lot of both experimental [18,47–48] and theoretical [49–52] attention in recent years. However, a general understanding of the dissipation mechanism is still lacking. Beside the electronic-structure effects [53–54] and adhesion hysteresis at the atomic scale [49,51], there is also a so called “apparent dissipation”. Recently Labuda et al. [55] showed that the apparent damping can be attributed to the transfer function of the piezo-acoustic excitation system. Therefore the dissipation signal needs to be carefully analyzed because it is one of the best indicators of the instrumental artifacts. As discussed in the previous section, the cross-talk is accompanied by the presence of a distinct dissipation signal.

Furthermore, the simultaneous measurement of the tunneling current and the frequency shift introduces additional complexity to the origin of the dissipation signal. Recently, Weymouth et al. reported a so called “phantom force” phenomenon [30], in which an additional force arises due to a limited electron transport of injected charge in samples with low conductance. However, not much is known currently about its impact on the dissipation signal.

**Figure 9:**
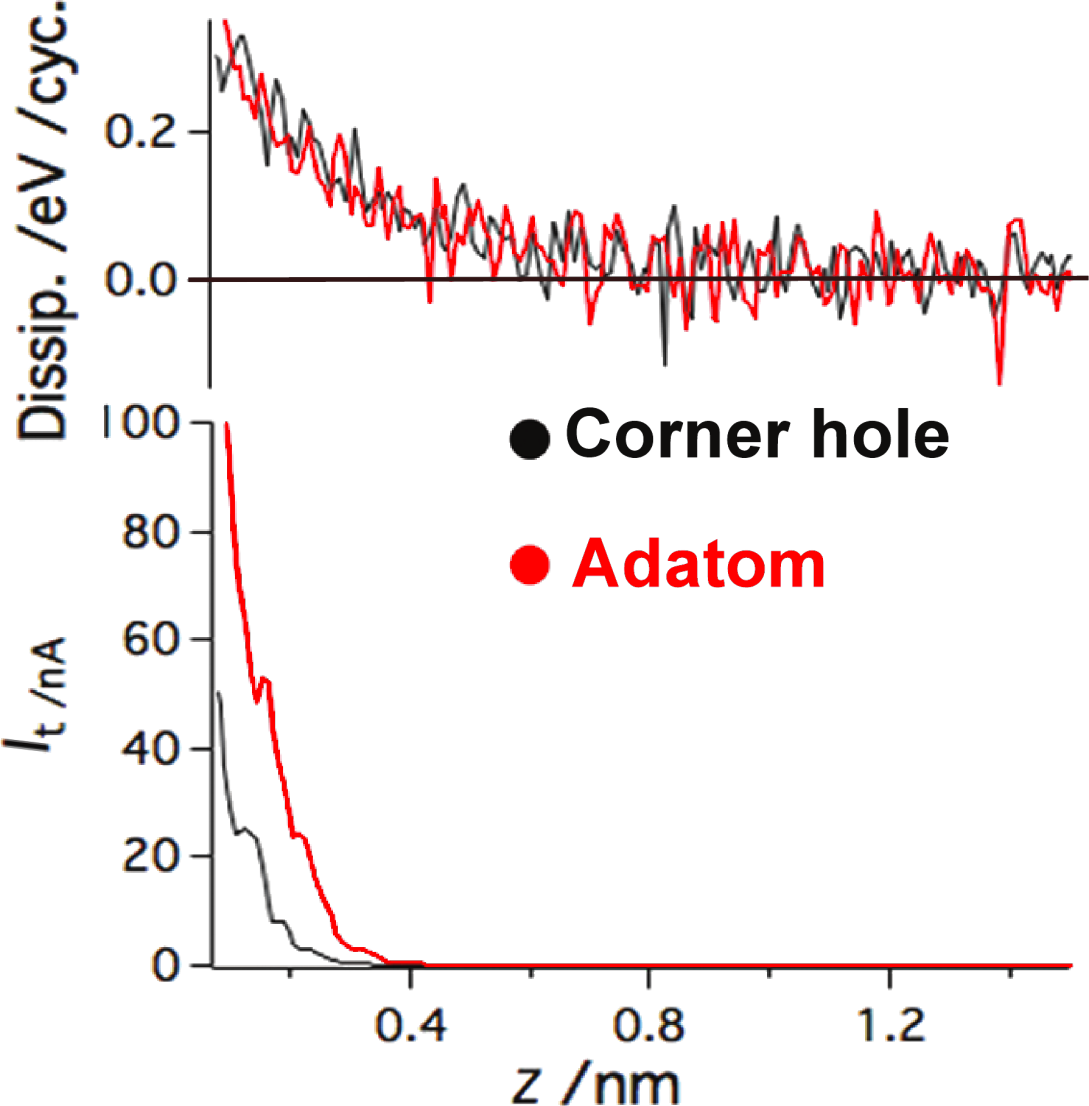
Analyses of the impact of the tunneling current on dissipation. It can be clearly seen that the tunneling current does not affect the dissipation, either directly (by cross-talk) or indirectly by induced nonconservative forces

In this section we analyze the effect of the tunneling current on the dissipation signal. This can be achieved by directly comparing the dissipation and tunneling current above the corner hole and adatom. As clearly shown in Figure 9 the dissipation signals are very similar, despite the strong difference in the magnitude of the tunneling current. Hence it can be concluded that the tunneling current does not directly affect (due to any kind of the cross-talk) the amplitude regulation in our modified experimental setup. One could also note that at room temperature the tunneling current does not give rise to any nonconservative forces in the case of the Si(111) 7×7 substrate.

In order to analyze the long-range dissipation in Figure 9, the relationship between the frequency shift and dissipation was investigated for both tip terminations presented in the previous subsection (reactive “tip A” and less reactive “tip B”). Comparing the frequency shift during the *z* approach for both tips, we can see that the the long-range forces are more dominant for “tip A”.

**Figure 10:**
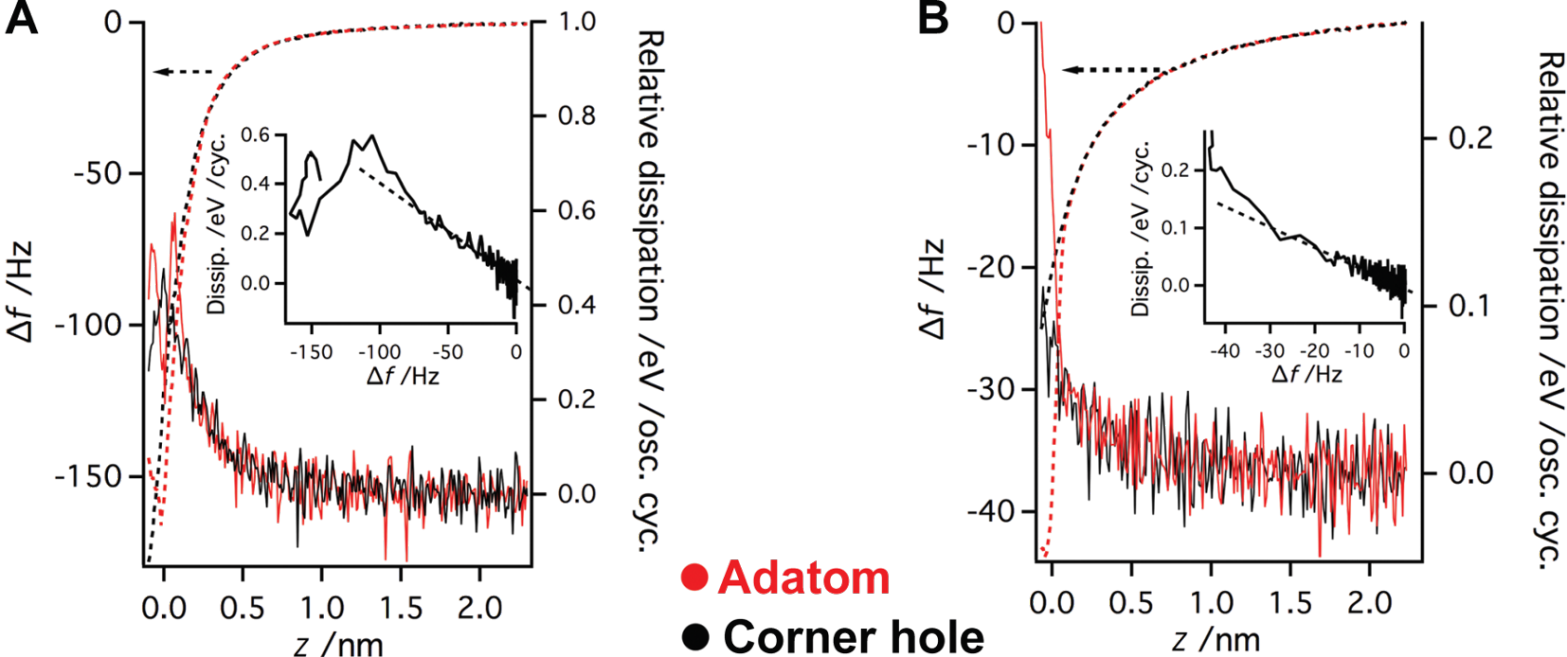
Relationship between the frequency shift and the dissipation for the reactive tip termination (A) and also for the less reactive one (B). Dissipation is plotted with a continuous line and the frequency shift with a dashed line. The corner hole data is marked with black and the adatom with red. In the insets the dissipation is plotted as a function of the frequency shift for the adatom data.

Interestingly we found that the long-range dissipation signal is correlated with the frequency shifts. In order to see the relationship between the frequency shift and the dissipation signal better, we plot the dissipation as a function of Δ*f* for data measured above the adatoms (see insets in Figure 10). In both cases, the long-range parts show a linear relationship. Furthermore, the slopes are nearly identical in both cases (4.0 ± 0.3) × 10^−3^ eV/Hz for “tip A” and (3.7 ± 0.6) × 10^−3^ eV/Hz for “tip B”. The proportional relationship is broken at −76 Hz in the case of “tip A” and −20 Hz in the case of “tip B”. The linear dependence between dissipation and Δ*f* suggests that the origin of the dissipation here is more instrumental (apparent) than related to the tip–sample interaction.

The apparent dissipation presented in our data can be explained by means of the effect of the piezo-transducer transfer function reported recently [55]. This idea is supported by the fact that the relationship between the frequency shift and the apparent dissipation in Figure 10 shows the same quantitative characteristics for both data sets. However, other tuning forks (operating at different eigenfrequencies) show different apparent dissipation or even no apparent dissipation at all.

**Figure 11:**
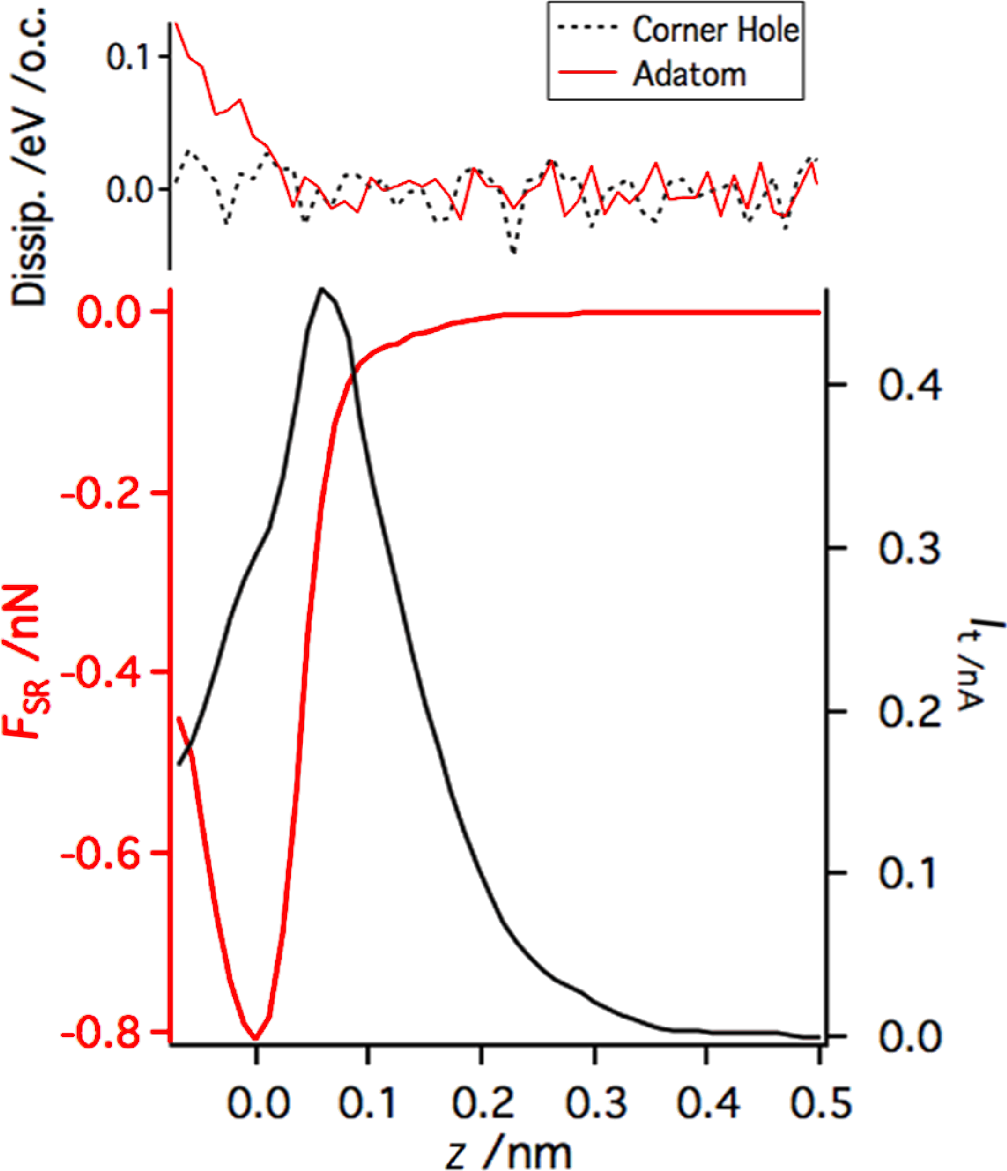
Corrected dissipation for damping measured above the adatom and above the corner hole. The corner hole shows only nondissipative interactions. The adatom starts to show dissipation when the *z* approach reaches and exceeds the force maximum.

Using the linear dependence of the apparent dissipation signal on Δ*f*, we can define a simple correction function independent of the surface site. Using the correction function, we can subtract the apparent dissipation signal from the data set. The bare short-range dissipation signal is plotted in Figure 11 together with the short-range interaction for better comparison. The same correction function was applied for damping measured above the corner hole as well. The dissipation signal becomes flat after the correction at large distances. A minor increase of the dissipation signal appears upon the onset of the chemical force above the adatom site. Therefore, we can attribute the origin of the dissipation signal to the adhesion hysteresis [49].

## Conclusion

We presented a modification of an Omicron qPlus VT system, designed to avoid crosstalk between the deflection and the tunneling-current channels. In the new design of the sensor, the current-to-voltage converter of the STM is connected directly to the tip with a gold wire. Beside separating the tunneling-current signal, it was necessary to replace the original ceramic support by a metal one in order to reduce the capacitive coupling between the channels. The site-specific force/tunneling-current measurements on the Si(111) 7×7 surface show excellent agreement with the published results obtained with an optical beam-deflection system. The sudden decrease of the tunneling current [46] caused by the formation of a covalent bond between the tip and the sample was clearly repeated, as in the previous work. Analysis of the dissipation signal shows that the tunneling current does not induce artificial damping up to 100 nA at room temperature. The dissipation detected by the amplitude regulator is the result of mainly two contributions. The first one, which has a long-range characteristic, is related to the instrumentation and can be subtracted. The second one appears only above the adatom site after the tip approach exceeds the position of the maximum of the short-range attractive force. We attribute the second contribution to the adhesion hysteresis [49].
